# Effects of a Self-Guided Transdiagnostic Smartphone App on Patient Empowerment and Mental Health: Randomized Controlled Trial

**DOI:** 10.2196/45068

**Published:** 2023-11-06

**Authors:** André Kerber, Ina Beintner, Sebastian Burchert, Christine Knaevelsrud

**Affiliations:** 1 Department of Clinical-Psychological Intervention Freie Universität Berlin Berlin Germany; 2 MindDoc Health GmbH Munich Germany

**Keywords:** patient empowerment, mental health–related self-management skills, help-seeking attitude, mental health literacy, internet-based interventions, unguided, self-guided, transdiagnostic mental health app

## Abstract

**Background:**

Mental disorders impact both individuals and health systems. Symptoms and syndromes often remain undetected and untreated, resulting in chronification. Besides limited health care resources, within-person barriers such as the lack of trust in professionals, the fear of stigmatization, or the desire to cope with problems without professional help contribute to the treatment gap. Self-guided mental health apps may support treatment seeking by reducing within-person barriers and facilitating mental health literacy. Digital mental health interventions may also improve mental health related self-management skills and contribute to symptom reduction and the improvement of quality of life.

**Objective:**

This study aims to investigate the effects of a self-guided transdiagnostic app for mental health on help seeking, reduced stigma, mental health literacy, self-management skills, mental health symptoms, and quality of life using a randomized controlled design.

**Methods:**

Overall, 1045 participants (recruited via open, blinded, and web-based recruitment) with mild to moderate depression or anxiety-, sleep-, eating-, or somatization-related psychopathology were randomized to receive either access to a self-guided transdiagnostic mental health app (MindDoc) in addition to care as usual or care as usual only. The core features of the app were regular self-monitoring, automated feedback, and psychological courses and exercises. The coprimary outcomes were mental health literacy, mental health–related patient empowerment and self-management skills (MHPSS), attitudes toward help seeking, and actual mental health service use. The secondary outcomes were psychopathological symptom burden and quality of life. Data were collected at baseline and 8 weeks and 6 months after randomization. Treatment effects were investigated using analyses of covariance, including baseline variables as predictors and applying multiple imputation.

**Results:**

We found small but robust between-group effects for MHPSS (Cohen *d*=0.29), symptoms burden (Cohen *d*=0.28), and quality of life (Cohen *d*=0.19) 8 weeks after randomization. The effects on MHPSS were maintained at follow-up. Follow-up assessments also showed robust effects on mental health literacy and preliminary evidence for the improvement of help seeking. Predictors of attrition were lower age and higher personality dysfunction. Among the non-attritors, predictors for deterioration were less outpatient treatment and higher initial symptom severity.

**Conclusions:**

A self-guided transdiagnostic mental health app can contribute to lasting improvements in patient empowerment. Symptoms of common mental disorders and quality of life improved faster in the intervention group than in the control group. Therefore, such interventions may support individuals with symptoms of 1 or more internalizing disorders, develop health-centered coping skills, prevent chronification, and accelerate symptom improvement. Although the effects for individual users are small and predictors of attrition and deterioration need to be investigated further, the potential public health impact of a self-guided intervention can be large, given its high scalability.

**Trial Registration:**

German Clinical Trials Register DRKS00022531; https://drks.de/search/de/trial/DRKS00022531

## Introduction

### Mental Health Problems Come With High Costs for Individuals and Society

More than 300 million people have depression and more than 250 million people are affected by an anxiety disorder around the world [1]. Population-wide studies in Germany have revealed that approximately 1 in 10 people meets the diagnostic criteria for depression. Approximately 1 in 5 lives with an anxiety disorder, and 1 in 20 people deals with chronic pain [2] or insomnia [3]. Depression and anxiety are among the top 10 contributors to health loss, accounting for 7.5% and 4.5% of years lived with a disability, respectively [1,4]. Mental disorders result in high direct and indirect costs, estimated at more than €60 (US $63) billion per year in Germany alone [5,6].

### Digital Mental Health Interventions May Reduce Symptom Burden

The efficacy of digital interventions has been shown for both the reduction of symptoms of common mental disorders and improvement of quality of life [7]. Most digital interventions address singular disorder categories and are structured similarly to disorder-specific treatment manuals [8]. These interventions consist of several subsequent sessions or modules. Interventions targeting the same disorder tend to be similar in terms of their components and content.

Guided digital interventions often yield larger effects than self-guided interventions (eg, the studies by Koelen et al [9], Lakhtakia and Torous [10], Moshe et al [11], and Schröder et al [12]), but a recent meta-analysis revealed that overall, the difference in effect sizes may not be very substantial [13], at least concerning anxiety disorders. Besides within-intervention guidance, guidance-related aspects of the study design such as the conduct of clinical interviews [14], use of automated reminders [15], or treatment setting in which the intervention is used [16] seem to be associated with greater effects. Nevertheless, although the scalability of web-based interventions with guidance is higher than that of traditional psychotherapy, it is limited because staff resources are required, even if the time allotted for guidance is limited. Self-guided interventions, although associated with only small to medium effects on individuals, can reach a larger number of patients and may thus have a larger public health impact [17].

### Comorbidity and Transdiagnostic Interventions

Comorbidity among mental disorders is high and has been shown to be largely associated with common causal pathways in several large studies [18,19]. Therefore, contemporary models of psychopathology postulate dimensional spectra (eg, internalizing, thought disorder, and externalizing) comprising multiple mental health syndromes with co-occurring genetic, neurobiological, environmental, and behavioral indicators [20]. Many pharmacological and psychological treatments yield transdiagnostic effects on multiple mental disorders [21-23]. Therefore, recent treatment protocols for mental disorders, such as the Unified Protocol [24] and the Common Elements Treatment Approach [25], replace disorder- and symptom-specific interventions with interventions that address common causal factors and have been shown to be transdiagnostically effective.

Most people with mental illness in Germany receive care exclusively from primary care physicians; 83% of patients with affective disorders (F3, International Classification of Diseases, 10th Revision) and 91% of patients with neurotic, stress, and somatoform disorders (F4, International Classification of Diseases, 10th Revision) do not receive treatment from a mental health specialist [26]. Differentiating between different mental disorders can be a challenge for primary care providers, especially given the high rate of comorbidity. The presence of emotional problems is recognized in most cases during contact with a primary care provider, but an accurate diagnosis is made much less frequently [27,28]. Thus, low-threshold transdiagnostic interventions may be more suitable than disorder-specific interventions in a primary care setting.

Digital interventions with a transdiagnostic approach have yet been less well researched than disorder-specific interventions, but the evidence base is growing (eg, the studies by Newby et al [29], Newby et al [30], and Păsărelu et al [31]). However, based on the findings discussed earlier, we expected a transdiagnostic digital intervention to impact a range of mental disorder symptoms, including anxiety and depression, as well as quality of life.

### Digital Mental Health Interventions May Reduce Treatment Barriers

Timely treatment for mental disorders is impeded by structural barriers, such as limited availability and high cost, but within-person attitudinal barriers may constitute an even stronger obstacle for treatment seeking. Wanting to handle the problem on one’s own, low perceived need for care, stigma, low knowledge about mental health services, and fear about the act of help seeking or the source of help itself have been shown to be the largest treatment barriers by far [32-34]. These factors reduce the chances of timely intervention and increase the risk of long-term symptom deterioration and chronification [35]. Low-threshold digital interventions that involve no personal contact and can be used anonymously can counteract at least some of these internal barriers and simplify help seeking.

An underinvestigated research area concerns the reduction of within-person barriers through digital mental health interventions. Some older studies evaluating digital mental health interventions for depression and anxiety have shown a decrease in self-stigmatization [36-38]. Effects on help-seeking attitudes and actual help seeking have been detected in some randomized controlled trials (RCTs) [38-41]. These effects seem to be linked to changes in health literacy [36,38]. In addition, a recent review on digital interventions found an increase in self-management behavior to be an important mediator of treatment effects [42]. However, research on the effects of transdiagnostic interventions on attitudinal barriers, help seeking, and self-management skills is lacking. A major advantage of transdiagnostic digital interventions in this respect is their potential to be truly low threshold. Unlike disorder-specific interventions, transdiagnostic interventions can be applied before diagnosis and, in a first step, help users determine whether they have a mental health problem at all, what their problem is, and whether they may need help from a mental health professional. Help seeking can then be actively encouraged by providing information and correcting unhelpful and false assumptions about mental health care.

### This Study

On the basis of the findings from previous research, we expected that the use of a self-guided transdiagnostic self-management app for mental health, in addition to care as usual (CAU), would lead to significant improvements in mental health literacy and variables that reflect patient empowerment, such as help seeking, reduced stigma, and self-management skills. If self-guided mental health apps have these effects, they would constitute a low-cost public health impact if made available for people with mental health problems in addition to CAU. Furthermore, we aimed to explore whether such an intervention leads to a greater reduction in symptoms of common mental disorders and a stronger improvement in quality of life than CAU only.

Thus, the aim of our study was to investigate whether the use of a self-guided transdiagnostic app for mental health is associated with improvements in mental health literacy and variables that reflect patient empowerment, such as help seeking, reduced stigma, and self-management skills. Furthermore, the intervention’s effects on symptoms of common mental disorders and quality of life were explored.

## Methods

### Ethical Considerations

This trial was registered in the German Clinical Trials Register (DRKS00022531**)**, and the local ethical committee of Freie Universität Berlin approved the protocol (AZ 039/2020).

### Design

To examine the effects of a transdiagnostic mental health app (MindDoc), we conducted a single-center RCT with 3 assessments [43]. We assigned participants to 2 groups in a 1:1 ratio. The intervention group (IG) received immediate access to the MindDoc app in addition to current care (CAU); however, a limitation exists in that we recruited participants without outpatient or inpatient psychotherapy at the start of the trial (see *Recruitment Strategy* section). In the control condition, participants were not given any guidance or encouragement to modify their current care and were informed that they had the option to receive access to the MindDoc app after the 6-month study period. Essentially, this created a waitlist control condition for the use of the MindDoc app. However, it is important to note that participants were allowed to use any treatments that were accessible to them during the trial.

Health literacy, patient empowerment, help-seeking attitudes, health service use, symptom distress, and quality of life were assessed before randomization (baseline assessment), 8 weeks after randomization (postintervention assessment), and 6 months after randomization (follow-up assessment).

The participants in the control group (CG) received access to the MindDoc app after completing their follow-up assessment.

### Intervention

The users in the IG received immediate access to the MindDoc app. The MindDoc app is a self-guided transdiagnostic intervention designed for individuals who want to take care of their mental health. It can be used across the mental health care spectrum, including for (indicated) prevention, early recognition, treatment, and aftercare. The core features of the app encompass regular self-monitoring and automated feedback and psychological courses and exercises.

The self-monitoring feature consists of an adaptive system of daily multiple-choice questions based on the Hierarchical Taxonomy of Psychopathology (HiTOP) [20] as well as regular mood ratings. On the basis of their entries in the self-monitoring feature, users receive regular automated feedback on relevant symptoms, problem areas, and in-app resources. Users also receive biweekly feedback on their overall mental health as well as encouragement to seek help depending on their health status.

The courses and exercises in the app provide information on common mental disorders and their treatment (psychoeducation) and teach self-management skills to support users in coping with symptoms and problems. Although some courses are disorder specific, most follow a transdiagnostic approach, considering the heterogeneity and high comorbidity in mental illness. The learning goals of the courses include, for example, identifying and gradually changing unhelpful thought patterns and basic assumptions, clarifying personal goals and values, promoting functional stress management behaviors, and fostering the ability to relax. All the courses and exercises are based on the fundamentals of cognitive behavioral therapy and its derivatives (eg, acceptance and commitment therapy and mindfulness-based stress reduction).

All the app’s content was developed by or under the supervision of licensed clinical psychological psychotherapists based on established approaches and guidelines for identifying and treating mental illness. The MindDoc app underwent no major changes in content or functioning during the intervention phase of the trial. Updates to the app that occurred during the research study were bug fixes and performance improvements and would not have impacted the therapeutic approach or usability of the app.

The app can send push notifications to a user’s phone every time a new question block is ready to be answered (3 times a day) and when automated feedback (insights) has been generated. Push notifications can be turned on and off by each user for question blocks, insights, or both. We did not monitor whether the trial participants used this feature. The notification settings in the RCT did not differ from those in routine application. There were no cointerventions, except for support by the study coordinator in installing the app.

The app contains detailed information on how to access mental health care. Participants who report a high symptom burden or functional impairment within the monitoring function of the app will be prompted to consult a health care professional in the automated feedback. Furthermore, users are repeatedly reminded that study participation does not substitute for diagnosis, counseling, or treatment by a licensed physician or psychotherapist.

The MindDoc app is a commercial product with both free and premium (paid) features. The study participants had free access to all the features. A more detailed description of the app is provided in the Multimedia Appendix 1. Descriptions of the app in this section and in Multimedia Appendix 1 correspond to the version of the app used in the research study and may not be exactly apply to the currently available version of the app.

### Recruitment Strategy

The participants were openly recruited via press releases and social media as well as health insurance member magazines and websites in Germany. Recruitment took place over a period of approximately 7 months (December 2020 to June 2021). Participation in the study was anonymous, but the participants were required to provide an (anonymous) email address through which they could be contacted.

### Participants and Procedures

#### Assessment Procedure

All assessments related to the trial were carried out outside the app via web-based surveys (self-assessment) on a web-based platform (Unipark/EFS Survey; Questback GmbH).

After receiving detailed written information about the study procedures and data processing, participants provided electronic informed consent. Participants were screened according to the predefined inclusion and exclusion criteria (see the subsequent sections). Eligible individuals then received access to the baseline assessment. Those who completed baseline assessments were randomly assigned to either the IG or CG in a 1:1 ratio using an algorithm provided by the assessment platform (Unipark/EFS Survey). They were immediately informed about the result of the assignment on the assessment platform and via email.

The participants in the IG received access to the MindDoc app and were recommended to use it for at least 8 weeks, although they had full access to it for 6 months. To this end, they received individual codes that unlocked the content of the app after downloading the app from the app store. The participants of both groups received an email invitation to the postintervention and follow-up assessments 8 weeks and 6 months after the baseline assessment, respectively. Participants who did not complete the postintervention or follow-up assessment were reminded 1 week later.

The participants were not financially compensated for participating in the study. However, participants who had completed the postintervention and follow-up assessments took part in a monthly raffle, where they could win a universal €50 (US $52.66) voucher that can be redeemed in a number of web-based stores.

#### Inclusion Criteria

We included adults with clinically relevant symptoms of internalizing disorders indicated by scoring above the cutoff for mild symptoms on one or more of the following scales: Patient Health Questionnaire–9 (PHQ-9) score>4 [44], Generalized Anxiety Disorder–7 (GAD-7) score>4 [45], Mini-Social Phobia Inventory score>6 [46], Patient Health Questionnaire–15 (PHQ-15) score>4 [47], Regensburg Insomnia Scale (RIS) score>12 [48], binge eating or compensatory behaviors>once/wk, BMI<18.5 kg/m^2^ or critical weight loss, and weight and shape concern.

In addition, participants needed to have full legal capacity (self-disclosure), have access to a smartphone (iOS [Apple Inc] or Android [Google LLC]) and the internet, and live in Germany.

#### Exclusion Criteria

We excluded individuals with severe symptoms of internalizing disorders (PHQ-9 score>19, GAD-7 score>15, or PHQ-15 score>14) and severely underweight individuals (BMI<15 kg/m^2^). We also excluded individuals who reported acute suicidality and individuals who reported a history of bipolar disorder, psychotic disorder, or substance use disorder. Participants who met these exclusion criteria were provided with detailed information on treatment options.

To ensure that the effects we discovered in the trial were attributable to the use of the app and not to other specific and intensive treatments, we excluded individuals with current or planned outpatient psychotherapy or inpatient treatment for a mental disorder. However, participants were allowed to use or initiate any treatment during the study period.

### Outcomes and Measures

#### Primary Outcomes

We assessed 4 coprimary outcomes in the trial: mental health literacy, mental health–related patient empowerment and self-management skills (MHPSS), attitudes toward help seeking (after 8 wk), and actual mental health service use (after 6 months).

Mental health literacy was assessed using the Mental Health Literacy Questionnaire, which is a 29-item scale with 4 dimensions (knowledge of mental health problems, erroneous beliefs or stereotypes, help-seeking and first-aid skills, and self-help strategies). The measure differentiates well between individuals with more experience with mental health and individuals with less experience with mental health and has good internal consistency (Cronbach α=.84) for the total score [49].

We used the Assessment of Mental Health Related Patient Empowerment and Self-Management-Skills questionnaire, which was constructed based on a systematic review on self-management skills for depression [50], a Delphi consensus study on self-help strategies for depression [51], 2 studies on useful self-management skills for mood [52] and anxiety [53] disorders from the patient perspective, and a conceptual framework for patient choice and empowerment in northern European health systems [54]. The questionnaire consists of 10 items in a statement format assessing patient empowerment based on how much patients agree or disagree on a 5-point Likert scale, for example, “I know well about the treatment options for my disease,” and 18 items in a question format assessing the frequency of self-management skills on a 5-point Likert scale, for example, “In the last 8 weeks, how often have you engaged in activities that gave you a feeling of achievement?” The complete Assessment of Mental Health Related Patient Empowerment and Self-Management-Skills can be retrieved in Multimedia Appendix 2.

Attitudes toward help seeking were assessed using the Inventory of Attitudes Toward Seeking Mental Health Services, which is a 24-item scale assessing 3 internally consistent within-person barriers to seeking mental health services: psychological openness, help-seeking propensity, and indifference to stigma. Internal consistency (Cronbach α=.87) and the validity of the assessment could be confirmed in separate samples [55].

Actual seeking of outpatient psychotherapeutic or psychiatric treatment was assessed via 2 questions asking whether these services were used in the last 6 months.

#### Secondary Outcomes

The PHQ-9 is the depression module of the self-administered version of the Primary Care Evaluation of Mental Disorders diagnostic instrument for common mental disorders. It scores each of the 9 Diagnostic and Statistical Manual of Mental Disorders, Fifth Edition (DSM-5) diagnostic criteria from 0 (not at all) to 3 (nearly every day). The PHQ-9 is a reliable (Cronbach α=.89) and valid measure of depression severity [44]. Higher scores indicate a higher symptom load.

The GAD-7 is a 1D instrument designed to detect symptoms of generalized anxiety disorder as defined in the DSM-5. The item scores range from 0 (not at all) to 3 (nearly every day). The GAD-7 is a valid and efficient tool for screening for anxiety disorders and assessing their severity in clinical practice and research [56]. Higher scores indicate a higher symptom load.

The PHQ-15 is the module for assessing the severity of somatic symptoms of the self-administered version of the Primary Care Evaluation of Mental Disorders diagnostic instrument for common mental disorders. It comprises 15 somatic symptoms from the PHQ, with each symptom scored from 0 (“not bothered at all”) to 2 (“bothered a lot”). The PHQ-15 is a reliable (Cronbach α=.80) and valid screening tool for somatization [47]. Higher scores indicate a higher symptom load.

The RIS [48] is a self-rating scale with 10 items for assessing the cognitive, emotional, and behavioral aspects of psychophysiological insomnia. It has good internal consistency with Cronbach α=.89 and distinguishes well between controls and patients with psychophysiological insomnia. Higher scores indicate a higher symptom load.

The Personality Inventory for DSM-5, Brief Form Plus is a short form of the Personality Inventory for DSM-5 with 34 items, which is compatible with the dimensional assessment of maladaptive personality expressions in the International Classification of Diseases, 11th Revision. The Operationalized Psychodynamic Diagnosis-Structure Questionnaire Short is a short 12-item measure for assessing the severity of personality dysfunction. Dimensional assessment of the severity and style of personality dysfunction according to DSM-5 and International Classification of Diseases, 11th Revision are important predictors of treatment course, adherence, and response and general psychopathology [57]. Both the Operationalized Psychodynamic Diagnosis-Structure Questionnaire Short (Cronbach α=.89) and the Personality Inventory for DSM-5, Brief Form Plus (average McDonald ω=0.81) are validated and reliable measures [58-60]. Higher scores indicate higher personality dysfunction.

Quality of life was assessed using the Assessment of Quality of Life-8 Dimensions, which is a 35-item self-assessment scale designed to evaluate health services that impact the psychosocial aspects of quality of life. It assesses 3 physical and 5 psychosocial domains of functioning. It has good reliability (Cronbach α=.96) and convergent and predictive validity [61]. Higher scores indicate a lower quality of life.

Secondary outcomes were symptoms of common mental disorders and quality of life 8 weeks and 6 months after baseline assessments.

To determine the overall burden of symptoms of common mental disorders, we calculated a composite score from the sum scores of the PHQ-9, GAD-7, PHQ-15, and RIS, divided by the respective scale span. The composite score calculated in this manner can take values between 0 and 1, with higher values indicating higher symptom burden.

All assessments were tested before fielding the trial; the web-based survey contained, on average, 12 items per page, and there was no adaptive testing. The questionnaires at the 3 assessment time points contained between 20 and 25 pages of questions, and all items were mandatory. We used no cookies or IP check but identified multiple entries from the same users through either multiple code redemptions on the same device or multiple participant code or email entries. We applied no weighting of the items, and there were no incomplete questionnaires, as all items were mandatory.

Use data were recorded directly in the MindDoc app. Data from the 2 sources (MindDoc app and study survey) were matched via a personalized download link, which users in the IG received after randomization. A day of use was recorded when a user actively engaged with the app on that day, such as answering questions or engaging with an exercise. An exercise was considered engaged with once it was opened.

### Statistical Analyses

Assumptions for the appropriate statistical tests were checked for normality through histograms, skewness, and Kolmogorov-Smirnov test; sphericity was assessed through Mauchly test; and the assumption of equality of variance-covariance matrices was investigated through Box test and Levene test.

If participants entered assessments several times, only the first assessment was used in the consecutive analyses. Participants were excluded if they fulfilled the exclusion criteria or were missing data from their baseline assessments. Missing data from the postintervention and follow-up assessments of nonbinary primary outcomes (mental health literacy, help-seeking attitudes, and MHPSS) were imputed using baseline scores on symptom severity, mental health literacy, patient empowerment, help-seeking attitudes, quality of life, and severity of personality dysfunction and demographic information by applying an iterative Markov Chain Monte Carlo method based on the initial treatment assignment. We calculated imputations for 10, 50, 100, 150, 200, 300, 400, and 500 iterations. Then, we calculated the fraction of missing information (FMI) index for all multiply imputed data sets. The FMI ranges from 0 to 1 (with 1 meaning that 100% of the data necessary for the planned inferences are missing) and is a reliable indicator of the validity of inferences based on imputed data sets [62]. For the following analyses, we chose the number of imputations that yielded no further decline in the FMI compared with the previous ones. To further investigate the robustness of the findings and address potential bias due to nonrandom missing outcome observations, we calculated Random Forest Lee Bounds (RFLBs) for all nonbinary primary and secondary outcomes as a second procedure to account for missing data and potential nonrandom missingness [63].

In addition, a per-protocol (PP) analysis was performed excluding participants who made the following protocol violations: (1) failure to download the app and complete the onboarding process, (2) use of the app before the randomization date, (3) reporting of the use of the MindDoc app during the intervention and follow-up periods in the waitlist condition, and (4) noncompletion of postintervention or follow-up assessment.

To further investigate the robustness of the results and avoid potential bias due to nonrandom missing outcome observations, RFLBs were determined based on the PP sample for all nonbinary primary and secondary outcomes. In this procedure, the first step uses the random forest (RF) procedure to determine variables based on the baseline data that are related to missing data at later measurement time points. On the basis of this, upper and lower bounds were calculated for the mean differences in actual values between the IG and CG on the respective measure under investigation. If the rate of missing values differs across the study arms, the RFLB procedure trims the outcome distribution of the group with the lower dropout rate by removing observations from the lower (upper) end of the distribution using RFs trained on baseline variables that are predictive of dropout so that an upper and a lower bound adjusted for potential nonrandom missing outcome observations are estimated for the treatment effect. Upper and lower bounds that do not include 0 indicate that the measured difference between the groups persists, that is, is robust, even under the assumption of systematic differences in dropouts (missing not at random) between the IG and CG. Baseline variables identified to be predictive of dropout using the RFLB procedure were further investigated for systematic differences between participants who did drop out at the postintervention assessment point and those who did.

To compare the intervention effects on all non–count-based primary and secondary outcomes, we applied analysis of covariance (ANCOVA) between the groups at the posttreatment and follow-up time points, adjusting for baseline scores both on the multiply imputed intention-to-treat (ITT) and the PP data set. Differences in mental health–related health service use between the IG and CG were assessed using chi-square tests for the available data and the PP sample at follow-up. Multiple imputation on zero-inflated count data, such as physician visits, yields unreliable estimates [64] especially if missing data rates are high. Concerns of multiple testing error for the primary outcomes were addressed through Bonferroni correction. Differences in count-based measures of health service use between the groups were assessed using chi-square tests in the PP sample.

To investigate the predictors of study dropout and adverse events, such as deterioration, baseline variables that were more predictive than a random variable in RF models were identified and investigated using 2-tailed *t* tests.

## Results

### Participant Flow

**Figure 1:**
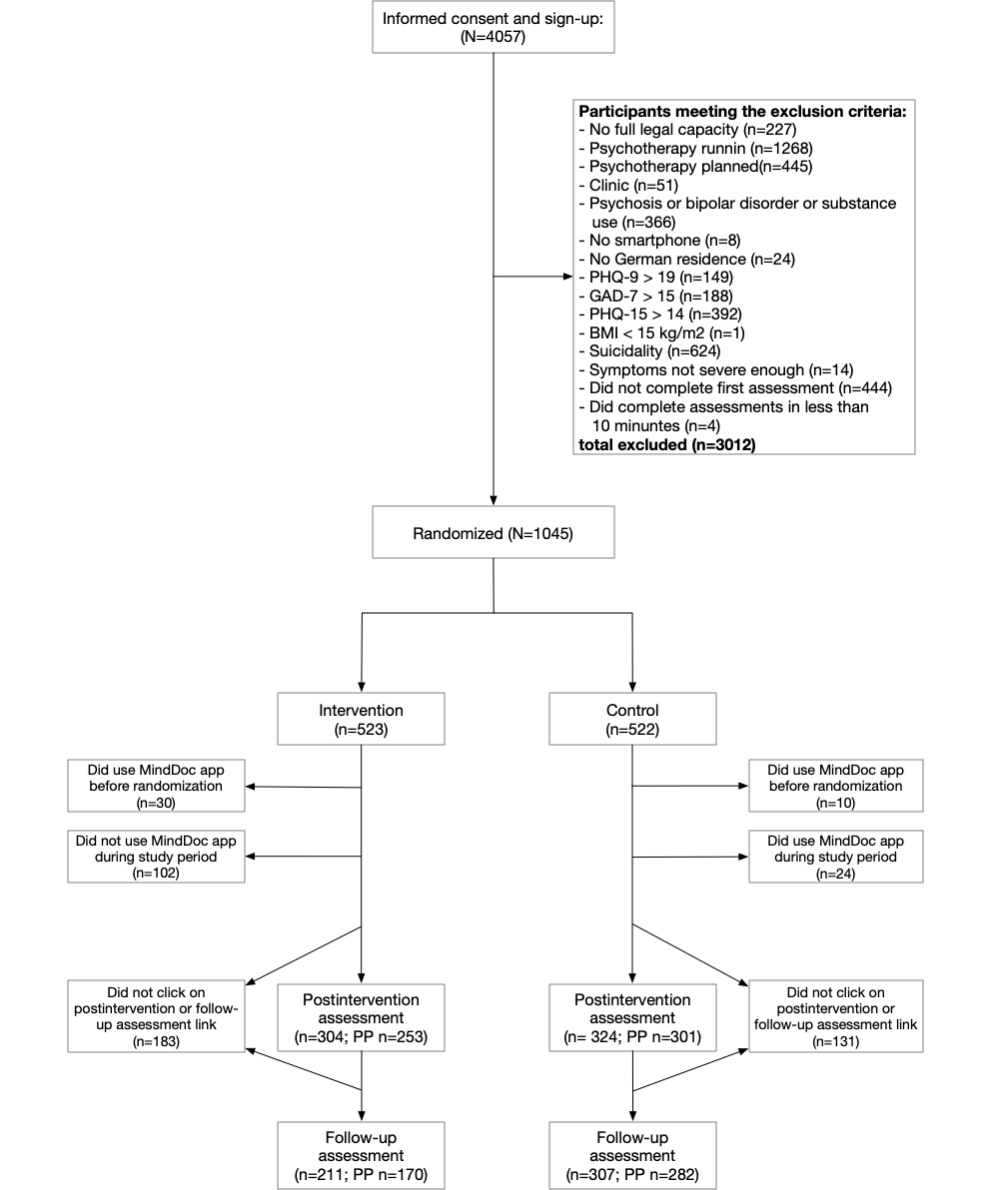
Participant flow. Note that owing to the possibility of only having 1 assessment after baseline, numbers after baseline assessments are not additive. GAD-7: Generalized Anxiety Disorder–7; PHQ-15: Patient Health Questionnaire–15; PHQ-9: Patient Health Questionnaire–9; PP: per-protocol sample.

Out of the 4057 individuals who provided informed consent to participate in the study, 1045 (25.76%) were randomized (Figure 1). Study dropout rates in the IG and CG were 41.9% (219/523) and 37.9% (198/522) at the postintervention assessment and increased to 59.7% (312/523) and 41.2% (215/522) at the follow-up assessment, respectively. Assessment data from participants without protocol violations (PP sample) was available of 48.4% (253/523) of the participants in the IG and 57.7% (301/522) of the participants in the CG at the postintervention assessment, decreasing to 32.5% (170/523) and 54% (282/522), respectively, at the follow-up assessment.

### Protocol Violations

The MindDoc app was available from the Apple App Store and Google Play Store during the study period. To decrease the risk of app use in the CG, we did not disclose which app was the subject of the trial until after randomization (IG) or follow-up (CG). Nevertheless, protocol violations related to app use did occur in the sample: of the 1045 participants, 40 (3.8%; IG: 30/523, 5.7%; CG: 10/522, 1.9%) used the app before randomization. This information was obtained based on self-report or the use logs of the app manufacturer. An additional 24 (4.6%) participants in the CG used the app during the study period. In the IG, 102 (19.5%) participants did not download the app or complete the app onboarding. The postintervention or follow-up assessment was not completed by 183 (35%) participants in the IG and 131 (25.1%) participants in the CG. Of the 1045 participants, 10 (1%) did multiple assessments, of which we only used the first assessment respectively for further analyses.

### Sample Characteristics

Table 1 presents the baseline characteristics of the participants. The mean age of the overall sample was 38.3 (SD 11.19; range 18-77) years. Most participants (769/1045, 73.59%) were female, and most (865/1045, 82.78%) had completed upper secondary education (“Abitur,” European Qualifications Framework level 4) or higher education.

Although current psychotherapeutic treatment was an exclusion criterion for the trial, almost 2 out of 3 (636/1045, 60.86%) participants reported previous psychiatric or psychotherapeutic treatment. The symptom burden was evenly distributed between mild and moderate in most of the individual symptom domains, and most participants (924/1045, 88.42%) reported elevated symptoms in ≥3 domains.

**Table 1:** Participant characteristics.

Characteristics		Full sample (N=1045)	Intervention (n=523)	Control (n=522)
**Sociodemographic characteristics**				
	Age (y), mean (SD; range)	38.3 (11.19; 18-77)	38.5 (11.65; 18-77)	38.21 (10.72; 18-71)
	Gender (women), n (%)	769 (73.6)	393 (75.1)	376 (72)
**Education, n (%)**				
	Higher education	371 (35.5)	188 (35.9)	183 (35.1)
	Upper secondary education	494 (47.3)	240 (45.9)	254 (48.7)
**Previous psychiatric or psychotherapeutic treatment, n (%)**				
	None	409 (39.1)	205 (39.2)	204 (39.1)
	One treatment provider	331 (31.7)	170 (32.5)	161 (30.8)
	Multiple treatment providers	305 (29.2)	148 (28.3)	157 (30.1)
	Inpatient treatment	234 (22.4)	118 (22.6)	116 (22.2)
**Depression**				
	PHQ-9^a^ total score, mean (SD)	10.62 (3.71)	10.62 (3.83)	10.62 (3.59)
	No or minimal symptoms (up to 4 points), n (%)	34 (3.3)	23 (4.4)	11 (2.1)
	Mild symptoms (5-9 points), n (%)	392 (37.5)	192 (36.7)	200 (38.3)
	Moderate symptoms (10-14 points), n (%)	448 (42.9)	216 (41.3)	232 (44.4)
	Moderately severe symptoms (15-19 points), n (%)	171 (16.4)	92 (17.6)	79 (15.1)
**Anxiety**				
	GAD-7^b^ total score, mean (SD)	8.26 (3.24)	8.25 (3.17)	8.27 (3.31)
	No or minimal symptoms (up to 4 points), n (%)	158 (15.1)	77 (14.7)	71 (13.6)
	Mild symptoms (5-9 points), n (%)	537 (51.4)	272 (52)	265 (50.8)
	Moderate symptoms (10-15 points), n (%)	350 (33.5)	174 (33.3)	176 (33.7)
**Somatic symptoms**				
	PHQ-15^c^ total score, mean (SD)	9.63 (3.24)	9.57 (3.24)	9.69 (3.25)
	No or minimal symptoms (up to 4 points), n (%)	139 (13.3)	68 (13)	71 (13.6)
	Mild symptoms (5-9 points), n (%)	428 (41)	220 (42.1)	208 (39.8)
	Moderate symptoms (10-14 points), n (%)	478 (45.7)	235 (44.9)	243 (46.6)
**Insomnia**				
	RIS^d^ total score, mean (SD)	14.04 (5.49)	13.98 (5.52)	14.10 (5.47)
	No or few insomnia symptoms (up to 12 points), n (%)	419 (40.1)	216 (41.3)	203 (38.9)
	Marked insomnia symptoms (≥12 points), n (%)	626 (59.9)	307 (58.7)	319 (61.1)
**Disordered eating, n (%)**				
	Binge eating at least once per wk in the last 3 mo	111 (10.6)	58 (11.1)	53 (10.2)
	Compensatory behaviors at least once per wk in the last 3 mo	49 (4.7)	27 (5.2)	22 (4.2)
	Underweight or significant weight loss in combination with weight and shape concerns	10 (1)	7 (1.3)	3 (0.6)
**Comorbidity, n (%)**				
	Elevated symptoms in 1 domain	35 (3.3)	20 (3.8)	15 (2.9)
	Elevated symptoms in 2 domains	86 (8.2)	45 (8.6)	41 (7.9)
	Elevated symptoms in ≥3 domains	924 (88.4)	458 (87.6)	466 (89.3)

^a^PHQ-9: Patient Health Questionnaire–9 (depression module).

^b^GAD-7: Patient Health Questionnaire–7 (anxiety module).

^c^PHQ-15: Patient Health Questionnaire–15 (somatic symptom module).

^d^RIS: Regensburg Insomnia Scale.

### App Use

**Figure 2:**
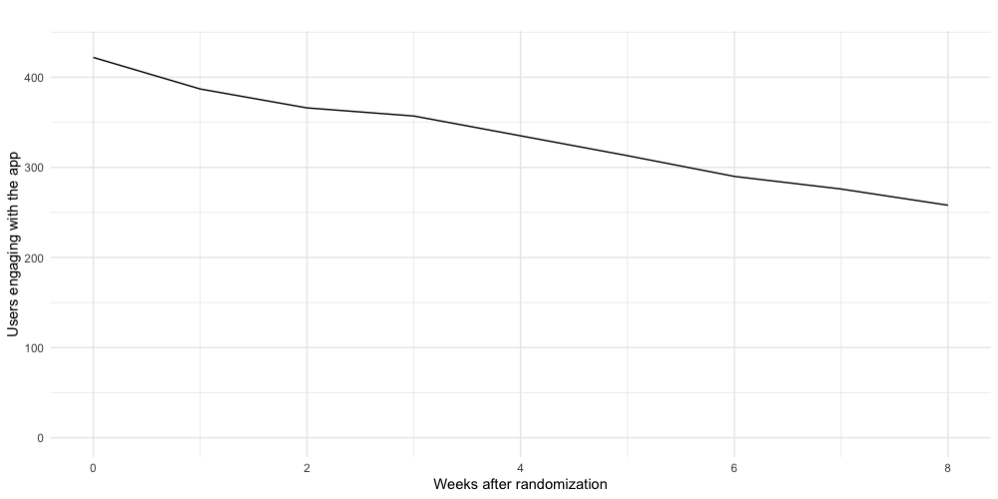
User engagements during study period.

The participants in the IG were recommended to use the MindDoc app for at least 8 weeks, but they were given unlimited access to the app for 6 months. Table 2 shows the use metrics of the participants in the IG. Figure 2 shows the number of active users per week during the intervention period. During this 8-week period, they used the app, on average, for 2 out of 3 days, and during the 6-month period, they used it for 4 out of 10 days. Although engagement with the questions continued well after the 8-week period, engagement with the courses and exercises subsided over time.

**Table 2:** App use metrics.

	All the participants in the intervention group who downloaded the app (n=423), mean (SD)	Participants in the intervention group without the protocol violation “app use before randomization” (n=393), mean (SD)
Days of use (within 8 wk)	38.4 (18.9)	38.5 (18.7)
Days of use (within 6 mo)	76.8 (61.6)	76.9 (61)
Question blocks answered (within 8 wk)	88.9 (53.3)	89.3 (53.2)
Question blocks answered (within 6 mo)	177 (159.4)	175 (157.8)
Exercises engaged with (within 8 wk)	16.2 (19.2)	16.3 (19.3)
Exercises engaged with (within 6 mo)	19.8 (24.5)	19.9 (24.6)

### Outcome Analysis

#### Primary Outcome Measures

The results of the efficacy analyses with respect to the 4 coprimary outcomes 8 weeks after baseline assessments are summarized in Table 3.

Intervention effects on the improvement in MHPSS could be confirmed 8 weeks after baseline assessments. ANCOVAs including baseline assessments as predictors showed significant (*P*<.001) results in both the multiply imputed ITT (100 imputations) and the PP analyses. Effect sizes for the between-group comparison for ITT were Cohen *d*=0.29 and Cohen *d*=0.28 for PP analyses 8 weeks after the baseline assessment.

Mental health literacy in the IG did not significantly exceed that in the CG 8 weeks after baseline assessments. Positive help-seeking attitudes in the IG did not significantly exceed those in the CG 8 weeks after baseline assessments. The proportion of participants who actually sought outpatient psychotherapeutic or psychiatric treatments was 35.5% (75/211; available data) or 33.5% (57/170; PP sample) in the IG and 25.7% (79/307; available data) or 25.5% (72/282; PP sample) in the CG. Results of between-group chi-square tests were *F*_1_=5.30 (*P*=.02) using available data and *F*_1_=2.95 (*P*=.09) only using data from the PP sample, indicating a trend toward higher outpatient treatment seeking in the IG, but the results were not significant in the PP sample.

Of the 4 coprimary end points, only the between-group differences in MHPSS yield a *P* value below the Bonferroni corrected α value of .013.

**Table 3:** Results of analyses of covariance (ANCOVAs) and Cohen d for the nonbinary coprimary outcomes (intention-to-treat [ITT] and per-protocol [PP] analyses) 8 weeks after baseline assessment^a^.

			Baseline assessment (IG^b^: n=523; CG^c^: n=522), mean (SD)		Postinterventi on assessment observed (PP; IG: n=253; CG: n=301), mean (SD)		Postintervention assessment estimated, mean (SD)		ANCOVA (ITT, multiple imputation)										ANCOVA (PP)										RFLB^d^			
									*F* test (*df*)			*P* value			Cohen *d* (95% CI)			*F* test (*df*)				*P* value			Cohen *d* (95% CI)			Lower bound^e^ (SD)			Upper bound^e^ (SD)	
**Mental health literacy (MHLq^f^)**										0.57 (99, 1130.75)			.45			0.01 (−0.13 to 0.16)				0 (1, 551)			.95			0.02 (−0.14 to 0.19)			−1.23 (0.69)			0.89 (0.6)
	Intervention	121.53 (9.12)		121.85 (10.72)		121.87 (11.64)																										
	Control	121.02 (9.17)		122.06 (8.48)		122.03 (8.71)																										
**Mental health–related patient empowerment and self-management skills (AMHPSS^g^)**										12.84 (99, 774.22)			<.001			0.29 (0.15 to 0.43)				13.29 (1, 155)			<.001			0.28 (0.11 to 0.44)			2.96 (1.86)			8.44 (1.56)
	Intervention	68.44 (18.44)		75.72 (19.05)		75.44 (19.54)																										
	Control	66.37 (18.95)		70.41 (19.48)		69.62 (20.46)																										
**Attitudes toward help seeking (IASMHS^h^)**										2.26 (99, 1012.95)			.13			0.12 (−0.02 to 0.26)				2.58 (1, 551)			.11			0.12 (−0.05 to 0.29)			−0.27 (1.05)			2.40 (0.73)
	Intervention	49.68 (9.19)		51.93 (9.59)		51.97 (9.59)																										
	Control	49.18 (9.95)		50.73 (10.14)		50.77 (10.42)																										

^a^The *F* test results were pooled using the D_2_ statistic [65].

^b^IG: intervention group.

^c^CG: control group.

^d^RFLB: Random Forest Lee Bound.

^e^These are lower and upper bounds of the Random Forest Lee Bound procedure.

^f^MHLq: Mental Health Literacy Questionnaire.

^g^AMHPSS: Assessment of Mental Health Related Patient Empowerment and Self-Management-Skills.

^h^IASMHS: Inventory of Attitudes Toward Seeking Mental Health Services.

#### Secondary Outcomes and Other Measures

For all secondary outcomes and other measures, including the mental health composite score and the measures for depression, anxiety, somatization, insomnia, and quality of life, the results showed significant between-group effects at the postintervention assessment point, controlling for pretreatment scores both in ITT and PP analyses (Table 4). Intervention effects ranged from Cohen *d*=0.19 (Cohen *d*=0.23 in PP sample) for quality of life to Cohen *d*=0.34 (Cohen *d*=0.39 in PP sample) for depression.

**Table 4:** Results of analyses of covariance (ANCOVAs) and Cohen d for the secondary and additional outcomes (intention-to-treat [ITT] and per-protocol [PP] analyses) at the postintervention assessment point^a^.

		Baseline assessment (IG^b^: n=523; CG^c^: n=522), mean (SD)	Postinterventi on assessment observed (PP; IG: n=253; CG: n=301), mean (SD)	Postintervention assessment estimated, mean (SD)	ANCOVA (ITT, multiple imputation)							ANCOVA (PP)							RFLB^d^		
					*F* test (*df*)		*P* value		Cohen *d* (95% CI)		*F* test (*df*)		*P* value		Cohen *d* (95% CI)		Lower bound^e^ (SD)			Upper bound^e^ (SD)	
**Symptom burden (composite score)**						13.75 (99, 633.44)		<.001		0.28 (0.13-0.43)		17.55 (1, 551)		<.001		0.34 (0.17-0.51)		0.02 (0.01)			−0.04 (0.01)
	Intervention	0.43 (0.07)	0.41 (0.09)	0.41 (0.09)																	
	Control	0.43 (0.07)	0.44 (0.09)	0.44 (0.10)																	
**Quality of life (AQoL-8D^f^)**						5.49 (99, 672.22)		.02		0.19 (0.05-0.34)		5.22 (1, 551)		.02		0.23 (0.06-0.40)		1.72 (1.60)			6.46 (1.26)
	Intervention	88.13 (13.77)	84.09 (16.36)	84.22 (16.67)																	
	Control	89.19 (14.49)	87.90 (16.92)	87.46 (16.92)																	
**Depression (PHQ-9^g^)**						21.54 (99, 690.83)		<.001		0.34 (0.19-0.49)		25.22 (1, 551)		<.001		0.39 (0.22-0.56)		1.13 (0.45)			2.32 (0.30)
	Intervention	10.62 (3.83)	8.96 (4.44)	9.10 (4.55)																	
	Control	10.63 (3.59)	10.71 (4.58)	10.65 (4.60)																	
**Anxiety (GAD-7^h^)**						8.73 (99, 693.01)		.003		0.22 (0.07-0.37)		11.34 (1, 551)		.001		0.28 (0.11-0.45)		0.68 (0.34)			1.61 (0.24)
	Intervention	8.25 (3.17)	7.31 (4.06)	7.39 (4.13)																	
	Control	8.27 (3.31)	8.46 (4.13)	8.30 (4.14)																	
**Somatic symptoms (PHQ-15^i^)**						7.07 (99, 837.46)		.008		0.20 (0.05-0.35)		2.14 (1, 551)		.008		0.24 (0.08-0.41)		0.46 (0.41)			1.62 (0.33)
	Intervention	9.57 (3.24)	9.02 (4.13)	9.07 (4.18)																	
	Control	9.69 (3.25)	10.05 (9.92)	9.92 (4.41)																	
**Insomnia (RIS^j^)**						5.31 (99, 672.22)		.02		0.19 (0.05-0.34)		5.22 (1, 551)		.02		0.23 (0.06-0.40)		0.33 (0.54)			1.89 (0.44)
	Intervention	13.98 (5.52)	12.65 (5.77)	12.64 (5.84)																	
	Control	14.10 (5.47)	13.71 (5.72)	13.52 (5.84)																	

^a^The *F* test results were pooled using the D_2_ statistic [65].

^b^IG: intervention group.

^c^CG: control group.

^d^RFLB: Random Forest Lee Bound.

^e^These are lower and upper bounds of the Random Forest Lee Bound procedure.

^f^AqOL-8D: Assessment of Quality of Life–8 Dimensions.

^g^PHQ-9: Patient Health Questionnaire–9 (depression module).

^h^GAD-7: Generalized Anxiety Disorder–7 (anxiety module).

^i^PHQ-15: Patient Health Questionnaire–15 (somatic symptom module).

^j^RIS: Regensburg Insomnia Scale.

#### Sensitivity Analyses

To further investigate the robustness of the results and avoid potential bias due to nonrandom missing outcome observations, RFLBs were determined for all nonbinary primary and secondary outcomes. Using this method, baseline variables that are predictive of dropout were identified, and participants from the lower and upper distributions on a specific outcome measure were excluded based on a RF decision tree trained on the group with more dropouts.

Differences between the groups in patient empowerment and self-management skills showed robustness with RFLBs excluding 0 for both postintervention and follow-up assessments.

For mental health literacy and attitudes toward help seeking, upper and lower bounds included 0 for the postintervention assessment, whereas RFLBs for the follow-up assessment did not include 0. Thus, it is highly unlikely that group differences in mental health literacy and help-seeking attitudes at follow-up are solely attributable to nonrandom missing outcome observations.

All secondary and additional outcomes showed robust treatment effects corrected for potential nonrandom missingness using the RFLB procedures at the postintervention assessment point.

#### Predictors of Dropout

Variable importance plots from the RF models indicating baseline variables that are predictive of attrition can be found in Multimedia Appendix 3. In the IG, 21 baseline variables were more predictive of dropout than a random variable. In the CG, 19 baseline variables were more predictive of dropout than a random variable. The 5 most predictive variables for dropout in the RF models in the IG were lower age; lower personality functioning; higher detachment; and lower mental health–related self-management skills, attitudes, and literacy. In the CG, lower mental health–related self-management skills, attitudes, and literacy; lower quality of life; lower age; and higher disinhibition were the most predictive variables in the RF models. We further investigated all variables identified to be predictive of dropout in the RF models using *t* tests for systematic between-group differences between participants who did and did not drop out at the postintervention assessment point. Here, we found small but significant differences in personality functioning (Cohen *d*=0.12), disinhibition (Cohen *d*=0.24), psychoticism (Cohen *d*=0.16), and age (Cohen *d*=0.15). That is, participants who dropped out were, on average, younger and had higher personality dysfunction scores.

#### Follow-Ups

Tables 5 and 6 show the results of ITT and PP ANCOVAs as well as RFLB analyses for all outcomes at follow-up. We found a robust group difference in mental health literacy (Cohen *d*=0.28, 95% CI 0.09-0.46) in addition to the difference in MHPSS that was maintained from the postintervention assessment point (Cohen *d*=0.34, 95% CI 0.18-0.55). The differences became apparent in the ITT, PP, and RFLB analyses. Attitudes toward help seeking yielded a small between-group effect (Cohen *d*=0.22, 95% CI 0.02-0.42) with RFLBs not containing 0, whereas ITT and PP ANCOVAs revealed a trend toward a group difference (ITT, *P*=.053; PP, *P*=.06). The mental health composite score showed a small between-group effect in the PP sample at follow-up (Cohen *d*=0.20, 95% CI 0.01-0.39), which could not be corroborated in RFLB analyses and ITT and PP ANCOVAs. From the measures of symptoms of common mental disorders, anxiety (GAD-7) showed a significant difference in the ANCOVA of the PP sample at follow-up with RFLBs not including 0, but the ANCOVA in the ITT sample did not reach significance (*P*=.34). Quality of life, depression, somatization, and sleep symptoms showed no significant between-group differences at follow-up (all *P*>.05).

**Table 5:** Results of analyses of covariance (ANCOVAs) and Cohen d for the nonbinary coprimary outcomes (intention-to-treat [ITT] and per-protocol [PP] analyses) at follow-up^a^.

			Baseline (IG^b^: n=523; CG^c^: n=522), mean (SD)		Follow-up assessment observed (PP; IG: n=170; CG: n=282), mean (SD)		Follow-up assessment estimated, mean (SD)		ANCOVA (ITT, multiple imputation)										ANCOVA (PP)										RFLB^d^				
									*F* test (*df*)			*P* value			Cohen *d* (95% CI)			*F* test (*df*)				*P* value			Cohen *d* (95% CI)			Lower bound^e^ (SD)				Upper bound^e^ (SD)	
**Mental health literacy (MHLq^f^)**										8.11 (99, 266.16)			.005			0.28 (0.09-0.46)				7.7 (1, 449)			.006			0.22 (0.03-0.42)			0.24 (1.40)			3.77 (1.25)	
	Intervention	121.53 (9.12)		123.73 (8.81)		124.13 (9.95)																											
	Control	121.02 (9.17)		121.66 (9.48)		121.38 (9.95)																											
**Mental health–related patient empowerment and self-management skills (AMHPSS^g^)**										12.13 (99, 0255.15)			.001			0.37 (0.18-0.55)				16.08 (1, 449)			<.001			0.38 (0.19-0.58)			3.51 (3.10)			11.33 (2.91)	
	Intervention	68.44 (18.44)		78.61 (19.31)		78.53 (21.64)																											
	Control	66.37 (18.95)		70.96 (20.33)		70.70 (21.29)																											
**Attitudes toward help seeking (IASMHS^h^)**										3.78 (99, 206.32)			.053			0.22 (0.02-0.42)				3.53 (1, 449)			.06			0.27 (0.07-0.46)			0.56 (1.56)			3.93 (1.56)	
	Intervention	49.68 (9.19)		53.05 (9.56)		52.51 (10.55)																											
	Control	49.18 (9.95)		50.56 (9.31)		50.34 (9.57)																											

^a^The *F* tests results were pooled using the D_2_ statistic [65].

^b^IG: intervention group.

^c^CG: control group.

^d^RFLB: Random Forest Lee Bound.

^e^These are lower and upper bounds of the Random Forest Lee Bound procedure.

^f^MHLq: Mental Health Literacy Questionnaire.

^g^AMHPSS: Assessment of Mental Health Related Patient Empowerment and Self-Management-Skills.

^h^IASMHS: Inventory of Attitudes Toward Seeking Mental Health Services.

**Table 6:** Results of analyses of covariance (ANCOVAs) and Cohen d for the secondary and additional outcomes (intention-to-treat [ITT] and per-protocol [PP] analyses) at follow-up^a^.

			Baseline (IG^b^: n=523; CG^c^: n=522), mean (SD)		Follow-up assessment observed (PP; IG: n=170; CG: n=282), mean (SD)		Follow-up assessment estimated, mean (SD)		ANCOVA (ITT, multiple imputation)										ANCOVA (PP)										RFLB^d^			
									*F* test (*df*)			*P* value			Cohen *d* (95% CI)			*F* test (*df*)			*P* value			Cohen *d* (95% CI)			Lower bound^e^ (SD)				Upper bound^e^ (SD)	
**Symptom burden (composite score)**										0.96 (99, 448.81)			.33			0.05 (−0.17 to 0.26)			17.55 (1, 449)			.06			0.20 (0.01 to 0.39)			0 (0.01)				0.04 (0.00)
	Intervention	0.43 (0.07)		0.40 (0.10)		0.42 (0.11)																										
	Control	0.43 (0.07)		0.42 (0.09)		0.42 (0.10)																										
**Quality of life (AQoL-8D^f^)**										0.71 (99, 371.03)			.40			0.06 (−0.15 to 0.26)			0.00 (1, 449)			.98			.06 (−0.13 to 0.25)			−2.73 (2.74)				4.49 (2.60)
	Intervention	88.13 (13.77)		84.32 (17.21)		84.12 (18.95)																										
	Control	89.19 (14.49)		85.39 (18.66)		85.21 (19.02)																										
**Depression (PHQ-9^g^)**										0.56 (99, 462.45)			.46			0.04 (−0.15 to 0.23)			2.09 (1, 449)			.15			1.15 (−0.04 to 0.34)			−0.20 (0.65)				1.64 (0.60)
	Intervention	10.62 (3.83)		8.95 (5.17)		9.47 (5.85)																										
	Control	10.63 (3.59)		9.72 (4.90)		9.66 (7.43)																										
**Anxiety (GAD-7^h^)**										0.89 (99, 448,48)			.34			0.06 (−0.13 to 0.26)			2.51 (1, 449)			.11			0.18 (−0.01 to 0.38)			0.03 (0.50)				1.37 (0.40)
	Intervention	8.25 (3.17)		6.99 (4.24)		7.43 (5.04)																										
	Control	8.27 (3.31)		7.76 (4.11)		7.72 (4.35)																										
**Somatic symptoms (PHQ-15^i^)**										0.98 (99, 401.19)			.32			0.07 (−0.13 to 0.27)			2.14 (1, 449)			.14			0.10 (−0.09 to 0.29)			−0.38 (0.59)				1.21 (0.58)
	Intervention	9.57 (3.24)		9.01 (4.06)		9.12 (4.51)																										
	Control	9.69 (3.25)		9.44 (4.40)		9.44 (4.64)																										
**Insomnia (RIS^j^)**										1.00 (99, 431.29)			.32			0.07 (−0.12 to 0.26)			0.06 (1, 449)			.80			0.02 (−0.01 to 0.21)			−0.93 (0.80)				1.24 (0.80)
	Intervention	13.98 (5.52)		13.25 (5.60)		13.05 (6.27)																										
	Control	14.10 (5.47)		13.39 (5.97)		13.49 (6.36)																										

^a^The *F* test results were pooled using the D_2_ statistic [65].

^b^IG: intervention group.

^c^CG: control group.

^d^RFLB: Random Forest Lee Bound.

^e^These are lower and upper bounds of the Random Forest Lee Bound procedure.

^f^AqOL-8D: Assessment of Quality of Life–8 Dimensions.

^g^PHQ-9: Patient Health Questionnaire–9 (depression module).

^h^GAD-7: Generalized Anxiety Disorder–7 (anxiety module).

^i^PHQ-15: Patient Health Questionnaire–15 (somatic symptom module).

^j^RIS: Regensburg Insomnia Scale.

### Adverse Events

Using the follow-up assessment, adverse events were recorded for individuals with available data (IG: 211/523, 40.3%; CG: 307/522, 58.8%). Suicidal ideations were reported by 48 (22.7%) participants in the IG and 75 (24.4%) participants in the CG (*χ*^2^_1_=0.1, *P*=.74). Symptom deterioration, that is, an increase in the scores of the GAD-7, PHQ-9, or PHQ-15 larger than minimally clinical important differences (MCIDs) that were available from previous validation studies [66-68] was detected in 19 (9%) participants in the IG and 31 (10.1%) participants in the CG for the PHQ-9 (*χ*^2^_1_=0.1, *P*=.79), 34 (16.1%) participants in the IG and 46 (15%) participants in the CG for the GAD-7 (*χ*^2^_1_=0.1, *P*=.82), and 22 (10.4%) participants in the IG and 33 (10.7%) participants in the CG for the PHQ-15 (*χ*^2^_1_=0.0, *P*>.99). Deterioration rates of the PHQ-9 scores after 8 weeks were 7.9% based on MCIDs (27.6% worsening of any size) in the IG and 13% based on MCIDs (44.4% worsening of any size) in the CG, with *χ*^2^_1_=3.8 and *P*=.05 for the group difference based on the MCID method and *χ*^2^_1_=18.5 and *P*<.001 for group differences based on any size of deterioration.

Variable importance plots from the RF models indicating baseline variables that were predictive of deterioration can be found in the Multimedia Appendix 3. In these models, 21 baseline variables were more predictive of deterioration than a random variable. Further investigation of all variables identified using *t* tests for systematic between-group differences between participants who did deteriorate at follow-up and participants who did not yielded significant differences in the GAD-7 (Cohen *d*=0.53), PHQ-15 (Cohen *d*=0.28), PHQ-9 (Cohen *d*=0.23), RIS (Cohen *d*=0.12), outpatient psychiatric or psychotherapeutic treatment (Cohen *d*=−0.31), other medical treatment (Cohen *d*=−0.24), and higher education (Cohen *d*=0.21). That is, participants who deteriorated had, on average, more severe symptoms at baseline; received less outpatient treatment before baseline; and had a higher education.

Inpatient treatment was reported by 43 (20.4%) participants in the IG and 55 (17.9%) participants in the CG (*χ*^2^_1_=0.4, *P*=.56). Severe health issues were reported by 29 (13.7%) participants in the IG and 45 (14.7%) participants in the CG (*χ*^2^_1_=0.0, *P*=.87). There were no serious adverse events that required a report to the institutional review board.

## Discussion

### General Findings

This study was the first to investigate the effects of a transdiagnostic self-guided mental health app on mental health–related patient empowerment, attitudes, self-management skills, and literacy in a large sample. Overall, the intervention yielded small but lasting effects on patient empowerment and self-management skills. It was also shown that improvements in symptom burden and quality of life can be accelerated by using the app. During the follow-up period, improvements in mental health literacy were detected, which were related to access to the intervention. Attitudes toward help seeking and use of outpatient psychiatric or psychotherapeutic services showed a trend toward change through intervention 6 months after baseline assessments. Although the effect sizes are small, the public health impact of the self-guided intervention can still be substantial given its scalability and comparatively low cost [17].

### Patient Empowerment, Mental Health Literacy, and Help Seeking

Previous research has shown that self-management skills play a key role in recovery from severe mental disorders [69-72]. Improving self-management skills and patient empowerment, especially in people with comorbid mental disorders, is an important factor contributing to mental health–related recovery processes [73]. Besides leading to more favorable treatment outcomes [74], improving self-management skills may lead to a sense of empowerment and responsibility as well as a sense of partnership between patients and clinicians [75].

The application of self-management skills may constitute an important component of interventions in multiple settings [76]. In an RCT with patients with chronic and treatment-resistant anxiety or depressive disorders, an intervention targeting self-management in addition to face-to-face treatment led to significant group differences in patient empowerment and social relations, and a larger proportion of patients were able to reduce treatment intensity [77]. A recent systematic review found self-help strategies to be an important mediator of outcomes in web-based interventions for depression [42]. Further, improving mental health–related self-management skills may shift patients’ approach to mental health problems from being disease centered to being more health centered [78].

Concerning attitudes toward help seeking and the use of outpatient psychiatric or psychotherapeutic services, we found some indications for intervention effects 6 months after baseline but no robust effects. As a substantial proportion of our participants (636/1045, 60.86%) had previous experience with psychiatric or psychotherapeutic treatment, internal barriers to treatment seeking were likely less prevalent in our sample than in the general population or other samples. To date, evidence on changes in help-seeking attitudes or behavior through web-based interventions is scarce. Although a more recent study found promising effects in a very small sample [38], findings from older studies with larger samples were mixed [37,39]. All these studies had a rather short follow-up period, especially given the fact that mental disorders can persist over years until those affected seek treatment. A longer intervention and follow-up period may be necessary to induce and measure changes in trust among mental health providers. A similar explanation may hold true for our findings on mental health literacy. Previous studies investigating changes in mental health literacy using follow-up measurements 6 months after baseline are scarce and not comparable in terms of sample size. Although a study on schizophrenia-related health literacy in a sample of 31 participants [79] found effects 6 months after a web-based psychoeducational intervention, another study [36] found comparable effects on depression and cognitive behavioral therapy literacy at the postintervention assessment point and 6-month follow-up. As participants in this study already had high mental health literacy scores at baseline, an interpretation of our findings may be that our participants were already quite mental health literate at baseline, and changes beyond this high average level of mental health literacy may take time.

### Symptom Burden and Quality of Life

Exploratory analyses revealed a small effect of the intervention on both symptoms of common mental disorders and quality of life at the postintervention assessment point. Symptom burden improved modestly during the intervention period in the IG but was slightly exacerbated in the CG. This pattern was observed in 3 (depression, anxiety, and somatic symptoms) out of 4 domains of symptoms, and the between-group difference was reflected in small effect sizes. Insomnia symptoms improved in both groups during the intervention period, but the improvement in the IG was greater. At follow-up, the results were mixed. On average, participants in the CG had caught up with those in the IG regarding improvements in both symptoms of common mental disorders and quality of life, although the mental health composite score and anxiety measure showed a trend toward greater change in the IG. Overall, both groups achieved comparable improvements during the combined intervention and follow-up period, but participants in the IG achieved these improvements earlier.

### User Engagement and Adverse Events

In participants in the IG who downloaded the MindDoc app, the average period of use went beyond the recommended 8 weeks. In addition, users frequently engaged with the intervention, on average, by accessing the app every other day.

There were no differences between the IG and CG in the frequency of adverse events (suicidal ideations, symptom deterioration, inpatient treatment, and severe health issues) 6 months after randomization. These findings are in line with those from other recent trials on digital mental health interventions that reported on adverse events (eg, the studies by MacLean et al [80], Baumeister et al [81], Oehler et al [82], Axelsson et al [83] and Reins et al [84]). Suicidal ideations were reported by 22.7% (48/211) and 24.4% (75/307) of the IG and CG participants who reported adverse events, respectively, during the follow-up period. Given the fact that more than half of the participants reported moderate or moderately severe symptoms of depression at baseline, this is an expected finding and corresponds with the prevalence of suicidal ideation in adults with major depression [85,86]. There was no indication that the adverse events were associated with the intervention and study participation. Symptom deterioration measured using the PHQ-9 after 8 weeks was significantly (*P*=.05 using MCIDs and *P*<.001 using any size of deterioration) higher in the CG and comparable with that in a meta-analysis from 2018 [86] investigating deterioration rates at the postintervention measurement point in RCTs of digital interventions for depression, although these investigated RCTs had more participant contact, such as weekly phone calls. Insignificant group differences in deterioration rates after 6 months and significant group differences at the postintervention measurement point were also found in a previous meta-analysis on digital interventions [87].

However, the IG in our study was associated with a faster improvement in symptoms of common mental disorders and quality of life than the CG. The MindDoc app showed effects that were comparable with findings from previous meta-analyses on the efficacy of self-guided app-based interventions for quality of life, depression, and anxiety [7]. Previous evidence on self-help interventions for somatization showed slightly higher effects [88,89]. Early symptom improvement is an important predictor of long-term outcome in mental disorders. In a study based on data of more than half a million health care users in England, the time passed without intervention was a strong and reliable predictor of later chronicity and nonresponse to treatment [90]. From this perspective, small but early improvement of symptoms through a self-guided web-based intervention such as the MindDoc app may prevent chronification and support remission and recovery.

As is common in studies investigating self-guided interventions [10], especially with web-based recruitment [91], dropout rates in our study were high. Moreover, the dropout rate in the IG was higher than that in the CG. This may be related to the fact that participants in the CG received access to the MindDoc app after the completion of the follow-up assessment, which could have represented an additional incentive that was absent in the IG. The most predictive variables for trial discontinuation in the IG were younger age and higher personality dysfunction. In particular, the latter is a relevant predictor of the amount of guidance required in psychological interventions [92]. Tackling this issue may require, besides human-delivered guidance, adjusting app behavior and content according to dimensional measures of personality functioning or other dimensional conceptualizations of psychopathology such as HiTOP [20]. The strongest predictors of deterioration within participants that did not drop out were more severe symptomatology and less outpatient treatment at baseline, which also point to the need for more guidance in cases with more severe psychopathology. Symptom severity was previously found to be an important indicator for more guidance in internet-based interventions [93,94] and higher treatment intensity in stratified care [95].

### Limitations

Nevertheless, this study had a number of limitations. First, dropout rates were high for both postintervention and follow-up assessments, which may have biased the findings, especially those of the ITT analyses using multiple imputation. However, provided sufficient auxiliary variables, multiple imputation can provide valid estimations even in situations with large proportions of missing data [62]. The dropout rates in our study were comparable with those in previous studies on self-guided internet-based mental health interventions [96], especially in studies with entirely web-based recruitment methods [91]. Further, there were no systematic differences in any primary or secondary outcome variable at baseline between participants who dropped out and those who did not drop out 8 weeks or 6 months after baseline assessments.

There was no active CG within the study design; however, participants were allowed to use any treatment during the study period, and a significant proportion of our participants stated that they had used mental health specialist care during the trial period.

Another limitation concerns the inclusion of participants based solely on self-report. Although this constitutes a very low threshold and a cost- and time-saving approach, it very likely resulted in the inclusion of a substantial proportion of participants with subclinical symptoms, which may have resulted in floor effects. Therefore, future research on the intervention should include clinical interviews upon the inclusion of study participants to confirm diagnoses.

Another limitation concerns the sample. Most participants were recruited through social media advertisements. This resulted in a study sample in which more than half of the participants had previous treatment experience. Therefore, mental health literacy at baseline was likely to be higher in this sample than in the general population or a primary care sample, and positive attitudes toward help seeking were likely to be more prevalent. Both may have resulted in floor effects on the respective outcomes. Therefore, future research on the effect of the intervention on mental health literacy and attitudes toward help seeking should specifically address treatment-naive participants and be conducted in a primary care setting.

### Conclusions

Using a nonguided transdiagnostic mental health app not only improved patient empowerment and self-management skills but also accelerated the improvement of symptoms of common mental disorders and quality of life. Thus, the intervention supported patients dealing with the symptoms of one or multiple internalizing disorders in developing more health-centered coping skills, preventing chronification, and facilitating recovery.

The effect sizes were small, but given the high scalability of a fully automated intervention, the impact on public mental health may still be considerable. With effect sizes comparable with those of antidepressants [97] and a possibly lower risk for unwanted somatic side effects, a self-guided app-based intervention can be a low-threshold, low-cost addition to mental health care services across the treatment spectrum.

Future studies are needed to specifically address the effects of self-guided mental health apps in various health care settings, including primary care, blended treatment, and aftercare, and differential effects in various groups of users, including those with subthreshold symptoms and those with full-syndrome mental disorders.

Another focus for future research may be the further customization of app behavior depending on dimensional measures of personality functioning or psychopathology, for example, adjusting the amount of automated in-app guidance and the number of automated in-app reminders, tailoring the user experience to meet the needs of users with different degrees of impairment, or providing more options for customization based on personal preferences.

## Appendix

Multimedia Appendix 1 Detailed description of the digital mental health app MindDoc.

Multimedia Appendix 2 Assessment of Mental Health Related Patient Empowerment and Self-Management-Skills.

Multimedia Appendix 3 Variables predictive of dropout or deterioration identified by random forest models.

Multimedia Appendix 4 CONSORT EHEALTH (Consolidated Standards of Reporting Trials of Electronic and Mobile Health Applications and Online Telehealth) checklist (version 1.6.1).

## Abbreviations

ANCOVA: analysis of covariance
CAU: care as usual
CG: control group
DSM-5: Diagnostic and Statistical Manual of Mental Disorders, Fifth Edition
FMI: fraction of missing information
GAD-7: Generalized Anxiety Disorder–7
HiTOP: Hierarchical Taxonomy of Psychopathology
IG: intervention group
ITT: intention-to-treat
MCID: minimally clinical important difference
MHPSS: mental health–related patient empowerment and self-management skills
PHQ-15: Patient Health Questionnaire–15
PHQ-9: Patient Health Questionnaire–9
PP: per-protocol
RCT: randomized controlled trial
RF: random forest
RFLB: Random Forest Lee Bound
RIS: Regensburg Insomnia Scale
