# Supplementary material for: Effects of a Self-Guided Transdiagnostic Smartphone App on Patient Empowerment and Mental Health: Randomized Controlled Trial
Source: JMIR Ment Health. 2023 Nov 6;10:e45068. doi: 10.2196/45068 (PMC10660244; doi:10.2196/45068)
Supplement: Multimedia Appendix 2 [file mental_v10i1e45068_app2.pdf]

Multimedia Appendix 2: Assessment of Mental Health related **Patient Empowerment and Self-Management-Skills (AMHPSS)**

Please rate the extent to which these statements apply to you.		Do not agree at all	Strongly disagree	Rather not agree	Rather agree	Strongly agree	Totally agree
1	I have read up on mental illness and its treatment on the Internet, in guidebooks, brochures or the like.						
2	I am well aware of the possible causes and triggers of my mental health problems.						
3	I am well aware of treatment options for my mental health issues.						
4	I can deal well with my mental problems.						
5	I know how to deal with emotionally difficult situations.						
6	I can judge well when I should consult a doctor or psychotherapist with my complaints.						
7	When it comes to taking medications, I make sure I get enough education and support from my doctor(s).						
8	If I don't understand something at a doctor's appointment, I will ask.						
9	I actively participate in decisions that affect my treatment.						
10	If I don't feel I'm in good hands with a doctor or psychotherapist, I talk about it						

	and look for someone else if necessary.						

The following questions refer to the last 8 weeks:		Never	Now and then	A little less than half the time	A little more than half the time	Most of the time	All the time
1	Did you make sure to get out of the house at least briefly each day?						
2	Have you been eating enough, regular and balanced meals to provide your body with energy, nutrients and vitamins?						
3	Have you done things that are good for you, even if you had to overcome yourself to do them?						
4	Have you made a point of incorporating positive activities into your daily routine?						
5	Have you been physically active, such as playing sports, hiking, Pilates, or gardening?						
6	Have you made sure to get regular and adequate sleep?						
7	Have they set out to do activities that can give you a sense of accomplishment?						
8	Have you talked about your problems with someone supportive in your social circle?						
9	Do you let friends or family know how you are feeling so they can relate to what you are going through?						
10	Have you been mindful of your sleep hygiene, such as avoiding						

	excessive media consumption or alcohol before bed, or making your bedroom as restful as possible?						
11	Have you kept in touch with friends or relatives who can help you get out the door and plan activities?						
12	Did you reward yourself when you reached a goal, even for small intermediate goals?						
13	Have you used relaxation techniques such as breathing techniques, progressive muscle relaxation or autogenic training?						
14	Have you used strategies for dealing with your mental health problems that have helped you in the past?						
15	Have you asked others for help, such as at work or around the house?						
16	Have you made sure to set realistic goals that are achievable in the short term?						
17	Have you meditated, used mindfulness or breathing techniques?						
18	Have you prayed, been to a place of worship, participated in spiritual rituals, or otherwise lived out your spirituality?						
