# Supplementary material for: Effects of a Self-Guided Transdiagnostic Smartphone App on Patient Empowerment and Mental Health: Randomized Controlled Trial
Source: JMIR Ment Health. 2023 Nov 6;10:e45068. doi: 10.2196/45068 (PMC10660244; doi:10.2196/45068)

Multimedia Appendix 3: Variables predictive for dropout or deterioration identified by random forest models

Figure 1: Variable importance plot for drop out at T2 in Intervention group. PF: Personality functioning; AMHPSS: Assessment of mental health related patient empowerment and self management skills; MHLq: Mental health literacy questionnaire; IASMHS: Inventory of Attitudes Toward Seeking Mental Health Services; PID: Personality inventory for DSM-5 brief form plus; HSU: Health service use; MINI-SPIN: Mini Social Phobia Inventory; rand: Random variable.

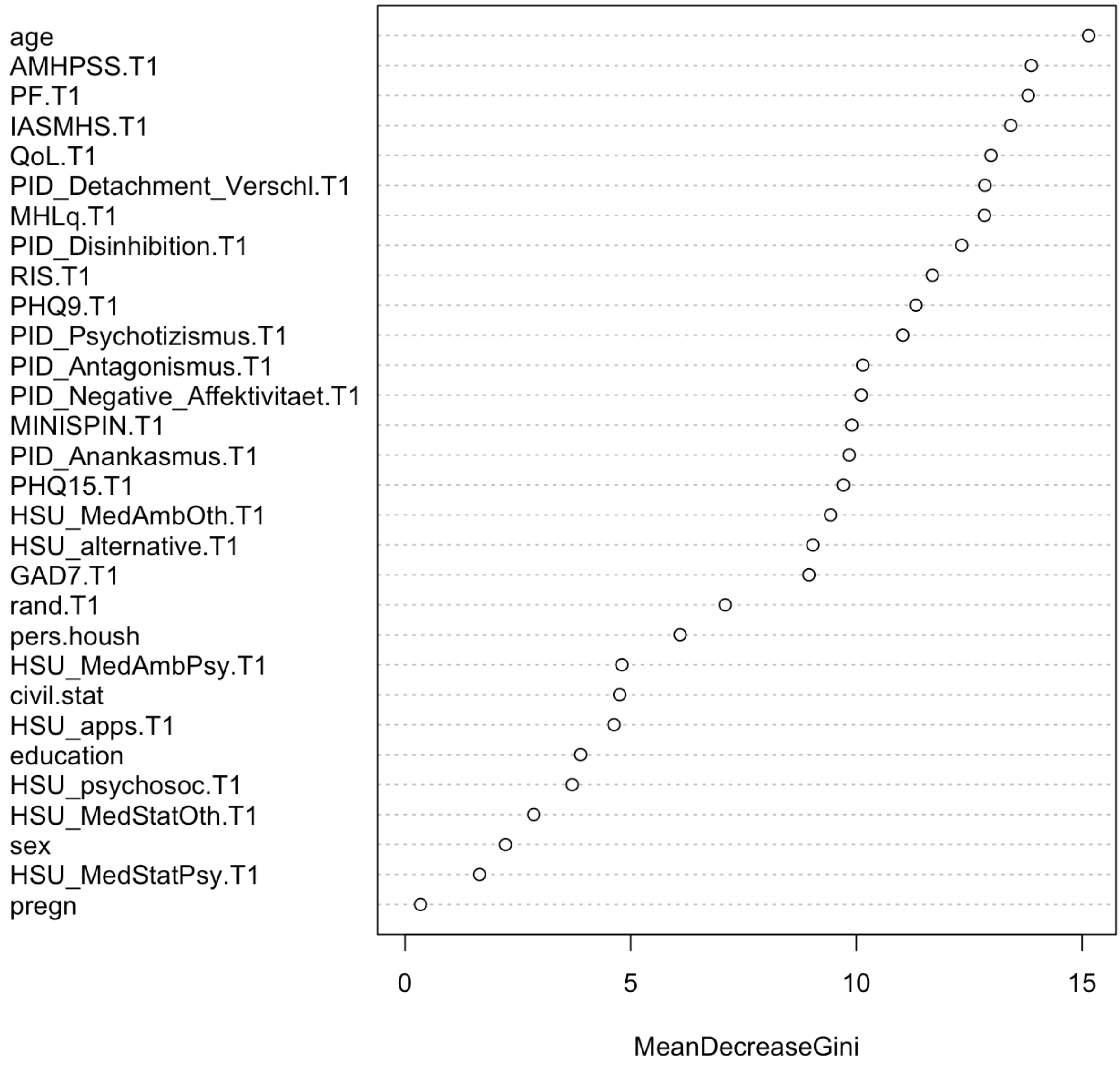

Figure 2: Variable importance plot for drop out at T2 in control group. PF: Personality functioning; AMHPSS: Assessment of mental health related patient empowerment and self management skills; MHLq: Mental health literacy questionnaire; IASMHS: Inventory of Attitudes Toward Seeking Mental Health Services; PID: Personality inventory for DSM-5 brief form plus; HSU: Health service use; MINI-SPIN: Mini Social Phobia Inventory; rand: Random variable.

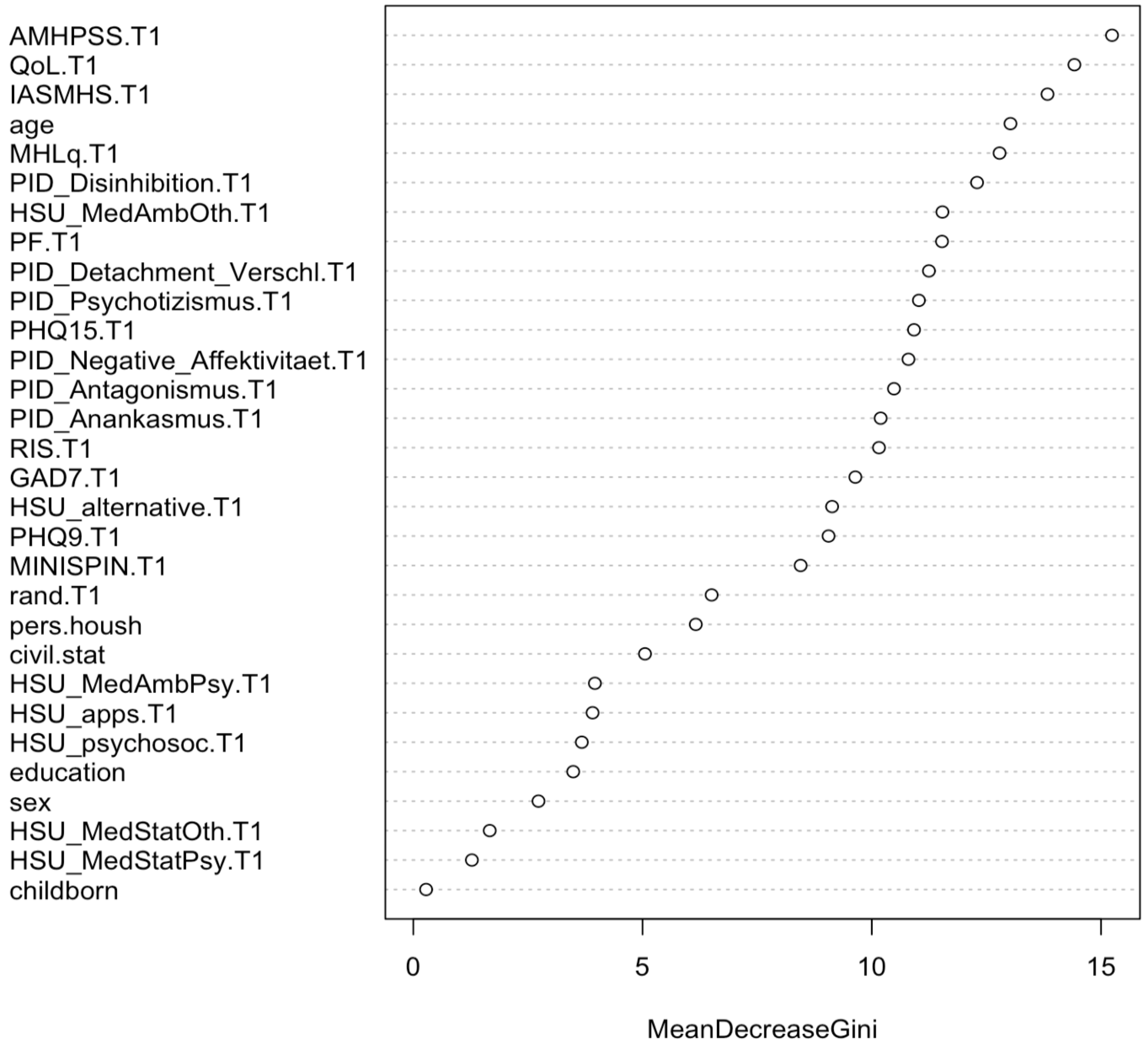

Figure 3: Variable importance plot for deterioration at T3.   PF: Personality functioning; AMHPSS: Assessment of mental health related patient empowerment and self management skills; MHLq: Mental health literacy questionnaire; IASMHS: Inventory of Attitudes Toward Seeking Mental Health Services; PID: Personality inventory for DSM-5 brief form plus; HSU: Health service use; MINI-SPIN: Mini Social Phobia Inventory; rand: Random variable.

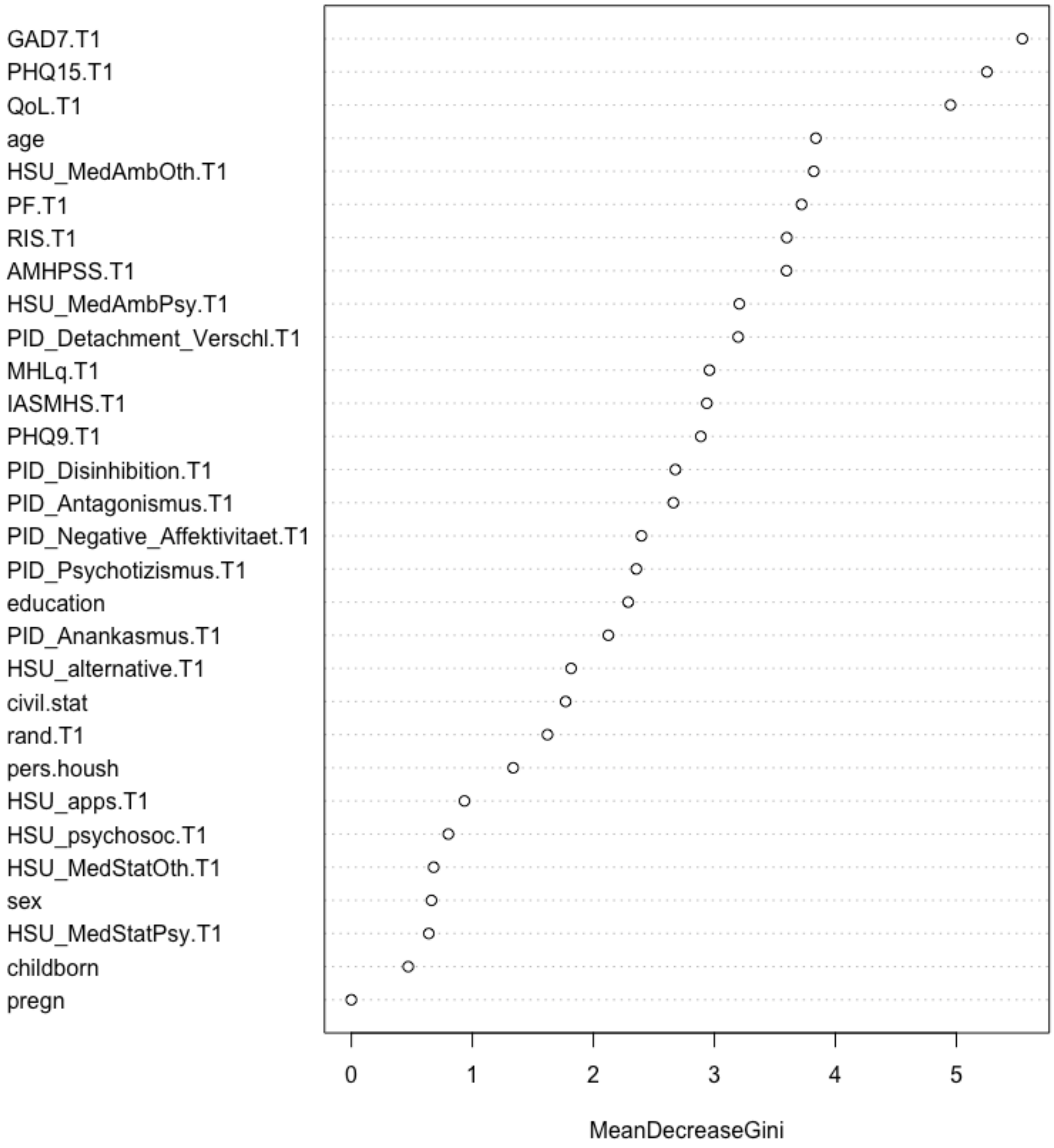

Supplement: Multimedia Appendix 3 [file mental_v10i1e45068_app3.pdf]
